# A Contribution to the Conservative Surgery of the Uterine Appendages

**Published:** 1903-09

**Authors:** James Swain

**Affiliations:** Professor of Surgery at University College, Bristol; Surgeon to the Bristol Royal Infirmary.


### A CONTRIBUTION TO THE CONSERVATIVE SURGERY OF THE UTERINE APPENDAGES.

BY

JAMES SWAIN, M.S., M.D. Lond., F.R.C.S.,

*Professor of Surgery at University College, Bristol;*
*Surgeon to the Bristol Royal Infirmary.*

---

To find it necessary to sacrifice healthy organs in order to effectually rid the patient of diseased ones must always be a matter of regret, and nothing can be more distasteful to the surgeon. Necessity knows no law, but wherever it is possible to conserve normal tissues the desirability of so doing needs little argument.

The modern treatment of compound fractures, myeloid sarcoma of the long bones, tuberculous joint disease, hammer-toe, and many other conditions, bears witness to the great advance which conservative surgery has made in dealing with many parts of the body; but the appreciation of what conservatism may do in affections of the uterine appendages is dawning more slowly.

The slavish adherence to an old-fashioned operative technique is largely responsible for the want of progress in that branch of pelvic surgery which forms the subject of this paper. It is still customary with many operators in dealing with tumours of the broad ligaments to regard salpingo-oöphorectomy as necessary for the removal of these conditions, with the result that normal tubes and ovaries are frequently—and quite needlessly—sacrificed.

Apart from the fact that it should be the highest aim of surgery to remove only diseased structures, it is not difficult to find good reasons for conservatism in regard to healthy Fallopian tubes and ovaries.

The distressing phenomena which so frequently accompany a menopause artificially induced, the mental effect on younger women of the knowledge that they have been "unsexed," and the possibility of conception if a wise conservatism has been followed, should all be borne in mind.

Moreover, there is a widespread belief that the ovaries produce an internal secretion necessary to the physiological well-being of the individual—a function analogous to that of the thyroid and other glands—and experiment has shown how greatly the metabolism of the body is affected by the loss of the ovaries. These ideas are supported by the clinical phenomena following castration, to which reference has just been made; and though conception is no longer possible after the final cessation of menstruation, it is still desirable to avoid the unnecessary sacrifice of the ovaries after the menopause, for it has not been shown that this function of internal secretion is even then in abeyance.

Much has already been accomplished in preserving healthy structures. Formerly it was the custom in cases of a diseased ovary of one side to remove the other (normal) ovary, lest the same disease should recur in it; now it is only in cases of sarcoma, carcinoma, and papilloma, where the opposite ovary is suspicious, that such a course would be countenanced. The removal of small "cystic ovaries" is now regarded by the best observers as unscientific and unjustifiable; adherent ovaries and tubes are simply released from their adhesions; the tube may be preserved in extra-uterine pregnancy, and ovaries affected with cysts of the corpus luteum are no longer sacrificed.

These facts, though not as well known as they should be, are well established, and there is no need to dwell on them; the points I desire to emphasize are connected with the removal of tumours of the broad ligament. It is in the manner of dealing with these growths that there is so much need of further knowledge and improvement, for their encapsulation has not been fully appreciated. We should have a smaller number of "incomplete ovariotomies" and of hysterectomies for "fibroma" of the broad ligament if the existence of a definite capsule was rightly understood.

I am not referring to those cases of uterine myo-fibromata and ovarian adeno-cystomata, which burrow into the layers of the broad ligament as a secondary development. These are fairly common, and in them conservatism is not usually possible. My remarks are limited to cysts and fibromata—or myo-fibromata—originating in the broad ligament itself.

It is desirable to consider exactly what is meant by the "capsule" which exists in these cases, but to prevent confusion reference must first be made to a "false capsule" which is not uncommonly found associated with ovarian tumours. This latter consists of adherent mesentery or omentum, or of inflammatory deposit. It is simply treated as an adhesion, and requires no further mention. The real capsules are provided primarily by the broad ligament, and to accurately grasp their anatomical arrangement we must understand the structure of the uterine appendages.

Above the level of the ovary the two layers of the broad ligament form a mesentery for the Fallopian tube (which is situated at the top of this portion of the broad ligament), and is known as the mesosalpinx. It is in this portion that cysts are found, whether they originate primarily in the broad ligament or burrow into it secondarily from the ovary. It contains the parovarium, and cysts arising in this structure form the commonest tumours enclosed in a capsule derived from the mesosalpinx.

Below the level of the ovary the two layers of the broad ligament are more widely separated, and here constitute the mesometrium. It is in this part of the broad ligament that fibromata or myo-fibromata will be found, whether they have developed *in situ* or represent a part of a myo-fibroma spreading out from the uterus. It is from the mesometrium that the capsule is derived in tumours of the lower part of the broad ligament.

The cellular tissue of the mesometrium is continuous with the retro-peritoneal connective tissue, so that large tumours can burrow extensively behind the peritoneum, which then constitutes a part of the containing capsule. Cysts originating in the mesosalpinx may invade the mesometrium and then burrow widely in the retro-peritoneal tissue as in one of the cases cited below (Case 3).

In addition to connective tissue, involuntary muscular fibres have been demonstrated in the mesometrium, so that there is no difficulty in understanding the pathology of the isolated myo-fibromata found encapsuled in this region.

In the process of expansion tumours of the mesosalpinx will be found with the Fallopian tube stretched across the top of the capsule, as in the ordinary parovarian cyst; whereas in tumours encapsuled by the mesometrium the layers of the meso-salpinx will be found to be unaffected on the top of the tumour.

In the right understanding of the various anatomical forms of capsule lies the secret of conservative surgery of the uterine appendages. The capsule is opened and the tumour is shelled out of its connective tissue bed, and thus ovary and Fallopian tube can be preserved intact. In the larger cysts a preliminary tapping is necessary.

After the enucleation of the tumour the treatment of the capsule is sufficiently simple: if healthy, it may be dropped back into the pelvis with or without sewing up the cut edges of the capsule. There is nothing to fear from the exudation of fluid or blood into the resulting cavity provided all vessels have been properly secured, and there is no need to cut away redundant parts of the capsule, which soon contracts. On the other hand, if the capsule is infiltrated with inflammatory deposits in association with a tumour which has suppurated, it will be wiser in some cases to attach the cut edges of the capsule to the abdominal incision and to drain the cavity. The need for this latter course is, however, extremely infrequent.

These remarks are well illustrated by a series of cases which have recently come under my care.

**Case 1. Cyst of Mesosalpinx.**—The patient, aged 27 years, had complained of pelvic pain for the past eighteen months. The pain was always worse at the menstrual periods, and during the past five weeks had been so severe as to necessitate the daily use of opium. The abdominal muscles were tense, and the patient indicated the left ovarian region as the site of greatest pain and tenderness. The uterus was normal in size and position. In the left broad ligament was a smooth globular swelling the size of an orange. The tumour allowed of a certain amount of movement, but it always retained the same relation to the uterus, and could not be moved out of the pelvis.

On opening the abdomen in the mid-line the swelling was found to be a smooth-walled, whitish cyst encapsuled in the upper part of the left broad ligament, with the Fallopian tube running over the top of it. The ovary was free. The mesosalpinx was incised over the tumour, which was cut into to allow of the escape of a clear, transparent fluid. The cyst-wall was then completely enucleated from the capsule formed by the mesosalpinx, and the hole in the broad ligament was closed with a few silk ligatures. No vessels were tied, and the ovary and Fallopian tube were preserved intact, except that a few small oöphoritic cysts were incised. The parietal wound was closed with silkworm gut, and the patient made an uninterrupted recovery.

Case 2. **Myo-fibroma of the Mesometrium.**—The patient, aged 34 years, had suffered from vaginal tenesmus and a sense of "weakness" for six years. For the past eighteen months sickness and pain had occurred at the menstrual periods. Three months ago there had been a very severe attack of hypogastric pain, and she had never been well since.

To the left of the uterus, and independent of it, a firm nodular swelling could be felt running out to the pelvic wall and more or less fixed. The tumour was the size of an egg, and a portion of it projected into Douglas's pouch. Some pain was produced when the tumour was firmly pressed upon. The uterus was normal in size and position. The right ovary was normal, but the left ovary could not be distinguished.

On opening the abdomen in the mid-line the tumour was found to be a myo-fibroma encapsuled in the layers of the mesometrium. The Fallopian tube, supported by an intact mesosalpinx, ran across the top of the tumour.

After incising the broad ligament the tumour was easily enucleated, and the hole in the mesometrium was closed with three silk sutures. Three small oöphoritic cysts in the left ovary were snipped and part of their walls removed, and a blood cyst from a recent Graäfian follicle in the right ovary was similarly treated. Both ovaries and tubes were preserved.

The abdomen was closed and recovery was uneventful. The tumour cut with a fibrous section, and microscopically showed the structure of a myo-fibroma.

Case 3. **Cyst commencing in the Mesosalpinx and burrowing into the Mesometrium and behind the Peritoneum.**—Three weeks before I saw her the patient, aged 42 years, had noticed a small tumour on the right side of the abdomen. A week later she went for a cycle ride, and afterwards felt very uneasy. Two days after this she was seized with great abdominal pain, chilliness, and retching, and the temperature was 103°. In about five days the pain had ceased and the temperature had come down to normal, but the tumour rapidly increased in size, and she frequently complained of faintness. The abdomen was distended, more on the right side than the left, by a smooth-walled, globular, fluctuating tumour which reached from the pelvis to half-way between the umbilicus and ensiform cartilage. The uterus was rather fixed, and some small myo-fibromatous nodules were felt in the cervix.

A 4-inch incision was made in the mid-line between the umbilicus and pubes. On separating a good many omental adhesions a cyst with red inflamed walls was disclosed. The tumour involved the right broad ligament, and reached up nearly to the liver by burrowing behind and between the mesentery and mesocolon, so that the intestine was implanted on the cyst and seemed part and parcel of its walls. On tapping the cyst about half a gallon of almost black fluid (altered blood) escaped, together with much soft, brownish fibrin loosely adherent to the walls of the cyst.

At first it appeared impossible to remove the cyst-wall, but the lower part was found to be closely applied to the uterus and to have originated in the broad ligament. The peritoneum and capsule of broad ligament were therefore divided over the tumour, and the whole cyst-wall was then, with some difficulty, completely enucleated from its bed. In doing this it was necessary to tie off many vessels and adhesions, but the ovary and tube were preserved intact. The cyst-wall was one-third of an inch thick and much inflamed. The abdomen was completely closed, and the patient made a rapid recovery.

This last was an extremely complicated case, and well illustrates the extensive retro-peritoneal burrowing of which many of these tumours are capable. Like the simple cyst in Case 1, it probably originated in the parovarium, but other cysts are found in the broad ligament, which appear to be of inflammatory or lymphatic origin,[1] and in such cases are frequently bilateral.

These inflammatory cysts are exemplified by two cases, which I will only briefly describe as the details have been published before.

**Case 4.**[2] **Multiple Intra-ligamentary Cysts.**—The patient, aged 29 years, had suffered from tuberculous peritonitis. After detaching some omental adhesions a cyst was found encapsuled in the right mesosalpinx. The capsule was divided over the cyst (which contained 6 ounces of a turbid greenish yellow fluid with cholesterin cystals in it), and this with three other cysts was enucleated from the right broad ligament. The

[1] See note at end of paper.

[2] "A Retrospect of a Third Series of Fifty Consecutive Intra-abdominal Operations," *Bristol M.-Chir. J.*, 1901, xix. 121.

enucleation left a cavity the size of a cricket ball, behind which the right Fallopian tube and ovary could be felt embedded in dense inflammatory tissue. A cyst as large as a pigeon's egg, and containing clear fluid, was enucleated from the left broad ligament in a manner similar to the removal of the cysts from the right broad ligament.

**Case 5.**[1] **Multiple Intra-ligamentary Cysts.**—The patient, aged 42 years, had two attacks of what appeared to be severe pelvic cellulitis, one of which nearly cost her her life, in the twelve months preceding operation. On opening the abdomen numerous and dense adhesions were found matting the appendix, uterus, and ovaries to the parietes. The vermiform appendix, which was diseased, was removed, and several cysts were enucleated from the right broad ligament. On the left side the ovary, Fallopian tube, and some broad ligament cysts were so densely matted that the mass was tied off and removed after it had been, with much difficulty, isolated from the dense adhesions surrounding it.

This last case was one of very great complexity and difficulty, and it is the only one in which a tube and ovary had to be sacrificed.

It is possible, too, that in larger myo-fibromata of the broad ligament than that mentioned in Case 2, the hemorrhage attending a rapid enucleation might be so severe as to endanger the patient's life. Under such circumstances it would be wiser to tie the ovarian and uterine vessels and the round ligament, and sacrifice the tube and ovary rather than run any great risk from hemorrhage.

Each case must be judged on its merits, but enough has been said to show how much can be done by enucleating cysts and solid tumours, and so preserving all normal structures.

Even where it is impossible to save the ovary and tube, a knowledge of the encapsulation which exists in broad ligament tumours is most valuable in the successful and rapid completion of the operation. Of such cases I could quote many examples, but it would be beside the purpose of the present subject.

In conclusion, a word may be said on the diagnosis of broad ligament tumours. They are fixed, more or less closely applied to the side of the uterus, and sessile because of their being

[1] "A Retrospect of a Further Series of Intra-abdominal Operations," *Bristol M.-Chir. J.*, 1902, xx. 33.

enclosed between the layers of the broad ligament. The myo-fibromata of the broad ligament occur at an earlier age than uterine myo-fibromata would be likely to be found burrowing into the mesometrium ; and from ovarian myo-fibromata or fibromata they are distinguished easily by the pedunculation and mobility which the latter tumours always present.

[Since writing the above, I have had a further case of double multiple broad ligament cysts, which throws some light on the pathology of some of these cases. The walls of the cysts contained a large quantity of unstriped muscle, and the inner surface was lined with columnar epithelium—forming papillomata in one instance. Their situation in the broad ligament, and the absence of ovarian tissue in the wall which contained so much muscular fibre, suggest that the cysts probably arose in some tubular structure, such as Gärtner's duct.—J. S.]

### A CASE OF PERFORATED DUODENAL ULCER, WITH REMARKS.

BY

G. MUNRO SMITH,

*Surgeon to the Bristol Royal Infirmary.*

